# Older Adults and Opioid Education: Lessons Learned From Experiences in Rural Arkansas

**DOI:** 10.1093/geroni/igaa057.177

**Published:** 2020-12-16

**Authors:** Robin McAtee, Leah Tobey, Corey Hayes, Laura Spradley, Sajni Kumpuris

**Affiliations:** 1 UAMS, Little Rock, Arkansas, United States; 2 University of Arkansas for Medical Sciences, Little Rock, Arkansas, United States; 3 University of Arkansas, Little Rock, United States

## Abstract

Nearly one-third of all Medicare participants were prescribed an opioid by their physician in 2015 (AARP, 2017) and in 2017, Arkansas had the 2nd highest opioid prescribing rate in the nation (CDC, 2019). Approaching older adults (OA) about opioids and pain management can be a sensitive topic. Educating and altering long-term treatment with opioids is especially challenging in rural areas where literacy, especially health literacy, is suboptimal. The Arkansas Geriatric Education Collaborative (AGEC) is a HRSA Geriatric Workforce Enhancement Program with an objective to improve health outcomes including an emphasis to decrease the misuse and abuse of opioids among older Arkansans. To address this crisis, the AGEC partnered with local leaders such as the AR Drug Director, academia, Department of Health and Human Services, and multiple community based organizations to create age-tailored educational programs. Unique aspects of approaching and educating rural OA about opioids and pain management will be reviewed. Outcomes will be discussed such as their lack of knowledge about: what is an opioid, why they were prescribed, and what are viable alternatives. Also discussed will be lessons learned that resulted in more effective methods of reaching and teaching rural OA. Partnering with the AR Farm Bureau helped the AGEC reach 100’s of farmers in the extremely rural and mostly agricultural areas. Learning to not use the word opioid resulted in more participants and in a more positive attitude and outlook on attempts to change the culture of opioid use, misuse and abuse among older Arkansans.

